# Expression and Prognostic Significance of Bcl-2 and Bax in The Progression and Clinical Outcome of Transitional Bladder Cell Carcinoma

**Published:** 2013-11-20

**Authors:** Bahram Golestani Eimani, Mohammad Hossein Sanati, Masoud Houshmand, Mitra Ataei, Fatemeh Akbarian, Naser Shakhssalim

**Affiliations:** 1 National Institute of Genetic Engineering and Biotechnology, Tehran, Iran; 2 Guilan University, Rasht, Iran; 3 Urology and Nephrology Research Center (UNRC), Tehran, Iran

**Keywords:** Apoptosis, Bcl-2, Bax, Bladder, Clinical Outcome

## Abstract

**Objective:**

To evaluate the mRNA expression ratio of Bcl-2/Bax both in normal and tumoral bladder tissues of patients with transitional cell carcinoma (TCC) of bladder and investigate potential correlation between this expression ratio and clinical outcome.

**Materials and Methods:**

In this experimental study, we used real time-PCR to investigate the expression of Bcl-2 and Bax both in normal and tumoral bladder tissues. The Bcl-2/ Bax expression ratio was determined in tumoral bladder tissues of patients with transitional cell carcinoma of the bladder (n=40) and correlation between expression ratios and the emergence of early relapses in a follow-up of 14-30 months was examined.

**Results:**

Relapse-free time in 14/31 patients (45.16%) with Bcl-2/Bax>1 was shorter than 9 months (range of 2-9 months) with 5.7 months average median while 17/31 patients (54.84%) with Bcl-2/Bax<1 are currently relapse-free (14-30 months). Bcl-2 and Bax expression levels were not solely correlated with clinical outcome and progression of carcinogenesis.

**Conclusion:**

The mRNA expression ratio of Bcl-2/Bax in tumoral bladder tissues may serve as a significant prognostic indicator in predicting the clinical outcome in low grade non-invasive bladder cancer.

## Introduction

Programmed cell death plays an important role in the cellular response to genotoxic stress; hence, loss of apoptotic response in tumor cells represents an effective mechanism involved in malignant progression and resistance to treatment (1).

Functional alterations in multiple genes involved in the control of cell division and cell death are thought to contribute to the rise of bladder cancer risk. Decreased rate of apoptosis provides tumor cells with selective growth advantage, facilitating neoplastic expansion. Tumor grade, being a traditional prognosticator, is not sufficiently reliable for accurate predicting of the clinical outcome of urothelial carcinoma. In order to investigate more precise indicators of biological aggressiveness, considerable attention has been paid for expression aberrations of apoptotic genes (2). Bcl-2 and Bax are two important regulator genes in the mitochondrial apoptotic pathway (3). The Bcl-2 gene product is thought to contribute to oncogenesis by suppressing signals that induce apoptotic cell death.

According to a number of researches (replace with studies. Research is usually not pluralised) high levels of Bcl-2 protein in a variety of solid tumors, including prostate carcinomas (4), colorectal cancer (5), squamouscell carcinomas of the lung (6), breast cancer (7) and nasopharyngeal malignancies have been shown (8).

Bax, an important homologue of Bcl-2, is a promoter of apoptosis. It has been proposed that the sensitivity of cells to apoptosis stimuli is closely related to the ratio of Bcl-2/Bax and other Bcl-2 homologues. When Bcl-2 is in excess, cells are protected. However, when Bax is in excess and Bax homodimers dominate, cells are susceptible to apoptosis (9). Recently, Bcl- 2 expression has been observed in urinary bladder tumors of 63% of patients with low grade disease; since Bcl-2 expression was found to be absent in normal adjacent bladder tissues, a hypothesis has been proposed that the expression of this gene may be correlated to a very early stage of bladder carcinogenesis (10).

The first objective of this study was to identify the role of Bax gene expression in the clinical outcome of low-grade bladder tumors expressing Bcl- 2 mRNA. A statistically significant correlation was found between the Bcl-2/Bax ratio and the clinical disease progression (r =14).

In the present study, transitional cell carcinoma of the bladder was treated by transurethral resection (TUR) and radical cystectomy. We investigated the relationship between Bcl-2 and Bax expression in the transcriptional level in bladder tumors and clinical outcome in patients with low-grade transitional cell carcinoma (TCC) of bladder.

In this study it is shown that the Bcl-2/Bax expression ratio reveals bladder carcinomas with a propensity for relapses, tumor grade and stage.

## Materials and Methods

### Specimens and patients

This experimental study comprises 40 patients with transitional cell carcinoma of the bladder. All patients were male and samples were obtained from tumor and adjacent normal tissues of each patient. All tumoral and normal samples were prepared from patients with noninvasive tumors during their first transurethral resection of tumor (TURT) without any other treatment. Sample collection was also undertaken for patients with high-grade tumors who followed their course of treatment by TURT or even radical cystectomy. The samples were obtained from the Urology and Nephrology Research Center (UNRC) at Shahid Labbafinejad Medical Center in Tehran, Iran. An ethical permission was issued by the UNRC Ethics Committee. All patients provided written informed consent. The research proposal for this study and the experimental steps were approved by the UNRC institutional review board.

The diagnosis of urothelial bladder cancer was confirmed due to histological analysis and patients were classified according to the tumor node metastases (TNM), pathologic staging and world health organization (WHO) grading system.

Samples were immediately frozen in liquid nitrogen and stored at -80˚C. All the patients had a follow-up urinary cytology for more than one year (14-30 months with 20.2 months ± 5.61 averages (every 3 months)).

### Quantitative PCR analysis

Total RNA was extracted from frozen tissues using RNX plus kits ( CinnaGen, Iran), according to the manufacturer’s instructions. The samples were eluted with 50 μl of RNase free water and stored at -70˚C. RNA concentration was determined using the spectrophotometry method. Complementary DNA (cDNA) was synthesized using RevertAid First Strand cDNA Synthesis Kit (Fermentas, Germany) following manufacturer’s protocol. Primers for detecting gene expression of Bax, Bcl-2 and β-Actin were designed by Beacon (Premier Biosoft, CA, USA), as listed in table 1.

Quantification of gene expression was done by RotorGene 6000 detection system (Corbett Research, Australia). PCR solution (20 μl) was composed of 2 μl cDNA, 4 μl of master mix solution of 5×HOT FIREPol® EvaGreen® qPCR Mix Plus Mix Plus kit (Solis Biodyne, Tartu, Estonia), 0.5 μl of each primer.

The thermal cycle parameters were: 1. cycle 95˚C for 15 minutes followed by 40 cycles at 95˚C for 10 seconds, annealing at 49˚C, 51˚C and 64˚C for Bax, β-Actin and Bcl-2 respectively for 20 seconds and 72˚C for 20 seconds (Table 2).

Amplification for each gene was performed separately. Standard curves for Bax, Bcl-2 and β-Actin were generated using serial dilution of cDNA. β-Actin was monitored as a reference gene and Bcl-2 and Bax expression levels were normalized with respect to β-Actin transcript and calculated by 2^-ΔΔCt^ method (11,12).

### Statistical analysis

Data were analyzed using the Statistical Package for the Social Sciences (SPSS) software (Advanced Statistics, version 17.0, Chicago, IL).

**Table 1:** Sequences of the primers used in real-time PCR assays


Name	5'-3'	Tm	PCR product size (bp)

**Bax-F**	GGTTGTCGCCCTTTTCTA	48.84	108
**Bax- R**	CGGAGGAAGTCCAATGTC	49.1	
**β-Actin-F**	GCGAGAAGATGACCCAGAT	50.87	88
**β-Actin-R**	GAGGCGTACAGGGATAGC	50.97	
**Bcl-2-F**	GATGTGATGCCTCTGCGAAG	65	93
**Bcl-2-R**	CATGCTGATGTCTCTGGAATCT	6	


**Table 2:** Protocol of real-time PCR


Fragments	Initial denaturation	Number of cycles	Denaturation	Annealing	Extention

**Bax**	95˚C-15minutes	40	95˚C-10seconds	49˚C-15seconds	72˚C- 20seconds
**Bax-R**	95˚C-15minutes	40	95˚C-10seconds	51˚C-15seconds	72˚C- 20seconds
**Bcl-2**	95˚C-15minutes	40	95˚C-10seconds	64˚C-15seconds	72˚C- 20seconds


**Table 3:** Expression ratio of Bcl-2/Bax mRNA evaluated by real time PCR and 2^-ΔΔCt^ method in bladder tumors


Patients	Stage	Grade	Relative expression of BCL-2/BAX	Time until relapse or disease-free(months)

**1**	pT1	Low	-	<3
**2**	pT1	Low	0.013	14
**3**	pT1	Low	0.001	14
**4**	pT1	Low	0.006	14
**5**	pT1	Low	0.09	14
**6**	pT1	Low	-	<3
**7**	pT1	Low	0.03	14
**8**	pT1	Low	0.05	14
**9**	pT1	Low	7.94	6
**10**	pT1	Low	-	<3
**11**	pT1	Low	2.35	5
**12**	pT1	Low	0.4	15
**13**	pT1	Low	2.3	9
**14**	pT1	Low	2.2	7
**15**	pT1	Low	0.19	14
**16**	pT1	Low	0.004	27
**17**	pT1	Low	-	<3
**18**	pT1	Low	-	<3
**19**	pT1	Low	0.37	26
**20**	pT1	Low	0.003	25
**21**	pT1	Low	0.27	26
**22**	pT1	Low	6.8	6
**23**	pTa	Low	0.001	14
**24**	pT1	Low	0.44	27
**25**	pT1	Low	-	<3
**26**	pT1	Low	0.18	18
**27**	pT1	Low	-	<3
**28**	pT1	Low	0.007	18
**29**	pT1	Low	0.04	30
**30**	pT1	Low	2.6	5
**31**	pT1	Low	-	<3
**32**	pT4	high	0.0005	18
**33**	pT3	high	0.003	16
**34**	pT1	high	1.5	5
**35**	pT2	high	4.85	8
**36**	pT1	high	6.15	4
**37**	pT1	high	3.1	7
**38**	pT1	high	3.9	6
**39**	pT2	low	2.7	7
**40**	pT1	high	-	<3


Patients whose tumoral tissue Bax gene expression was not detected by real-time PCR.

## Results

The age of the cases varied from 49 to 83 with 62 years mean and 7.16 standard deviation. In normal tissues, while Bcl-2 was not found to be expressed, Bax was present in 100% of samples. All tumoral tissues showed expression of Bcl-2, but in 9/40 (22.5%) of these tissue samples no detectable expression levels of Bax gene was observed (Table 3).

### Bax expression ratio in tumoral/normal tissue of patients

On the basis of Bax expression ratio in tumoral/ normal tissue (BaxT/BaxN), patients were divided into 2 groups. The first group with a BaxT/BaxN >1 was 19/40 (47.5%), and the second group with a BaxT/BaxN<1 was 21/40 (52.5%). Among the first group, 5/19 (26.3%) patients had high-grade tumors and 14/19 (73.7%) had low-grade tumors. In the second group, 4/21 (19.05%) patients had high-grade tumors and 17/21 (80.95%) had low-grade tumors.

Bcl-2 and Bax expression level were not solely correlated neither with the emergence of relapses nor with any of the known prognostic variables, such as stage and grade.

Because the experimental evidence suggests that the balance between antiapoptotic and proapoptotic members of the Bcl-2 family is a much better determinant of the sensitivity to apoptosis, we evaluated the Bcl-2/Bax expression ratio, instead of each individual expression pattern. The correlation evaluation between Bcl-2/Bax expression ratio in tumoral tissues and known prognostic variables showed that 77.8% of patients with high-grade tumor had Bcl-2/ Bax ratio >1 and only 22.2% of patients with low-grade tumor had the ratio more than one, thus, we observe the significant association between Bcl-2/Bax expression ratio and histological grade (p<0.05). But it is important to notice that there is no significant association between this ratio and the clinical stage of tumors (p=1.003).

### Evaluation of dependence between Bcl-2 and Bax expression and relapse-free time of patients

In this part of study we investigated 31 patients with primary low grade non-invasive bladder cancer, whose tumoral tissue samples were obtained from transurethral resection of bladder tumor (TURBT). It is shown that the Bcl-2/Bax expression ratio reveals bladder carcinomas with a propensity for relapses, regardless of tumor grade and stage. When we evaluated dependence between each gene expression and relapse-free time of patients in the first 30 months follow-up (mean follow- up period 20.2 ± 5.61 months), we found that high Bcl-2/Bax expression ratio, but not Bcl-2 and Bax expression separately, reached statistical significance in the prediction of relapses. On the basis of the Bcl-2/Bax ratio in tumoral tissues, patients were divided into 3 sub-groups, the first being characterized by Bcl-2/Bax ratio >1 (21/40; 52.50 %), the second by Bcl-2/Bax <1(10/40; 25%) and the last by the lack of Bax expression (9/40; 22.50%). The median relapse-free time in the first group of patients was 6.3 versus 18.5 months for the second group and it was less than 3 months in patients of the third group. When Bcl-2/Bax ratio was taken into account, patients with a high Bcl-2/Bax ratio had significantly shorter recurrence-free intervals than did patients with a low Bcl-2/Bax ratio (p<0.05).

## Discussion

This study was conducted to determine whether mRNA levels of apoptosis-regulators (Bcl-2 and Bax) and Bcl-2/Bax expression ratio in tumoral tissues could serve as independent parameters to envisage clinical outcome for patients with TCC of bladder. The ratio of Bcl-2 to Bax as its inhibitory homologue has been considered to be more important biologically in bladder cancer than Bcl-2 expression alone (10).

Our study showed that Bcl-2 and Bax expression levels were not solely correlated with either the emergence of relapses or any of the known prognostic variables such as stage and grade.

Overexpression of Bax plays an important role in apoptosis (13). Bax suppresses Bcl-2 expression and the extent of p53 suppression of Bcl-2 expression may be tissue-specific. Overexpression of Bax accelerates apoptotic death and Bax also counters the cell death repressor activity of Bcl-2 (14).

Early relapse in some of the tumors of the urinary bladder happens quite often clearly indicating the existence of heterogeneity in such malignancies. The forecast of their biological behavior solely on the basis of pathological parameters such as size, histological type and grade of differentiation is frequently complicated for clinicians. In fact, it is not rare to observe quicker change toward metastatic invasion in some forms of superficial bladder cancer independently of the clinical staging (10).

Despite improvements in survival rates, there is still a serious clinical problem related to the identification of patients with tumors at high risk for more rapid progression and metastatic invasion. The amplification and rearrangement of some oncogenes are known to regulate the progression of superficial bladder tumors towards invasive and metastatic forms (15).

The suggested hypothesis supposes that altered pathways of cell death may contribute to the very early stage of disease. Particularly, Bcl-2 over-expression was revealed to be a frequent molecular event involved in the first stage of bladder carcinogenesis (16).

In this study we analyzed the expression of two genes involved in apoptotic pathways (Bcl-2 and Bax), in order to shed light on their interaction during the bladder carcinogenesis, and their impact on clinical outcome.

Among the 31 patients examined, a group of 12 displaying a similar expression pattern (characterized by Bcl-2/Bax expression ratio> 1) was identified. Relapse-free time in all of these patients ranged between 4-9 months. In the other group, where the expression pattern showed a Bcl-2/Bax expression ratio<1, relapse-free time appeared to be considerably higher and all patients are currently healthy. Consequently, higher level of Bax expression in tumors compared with Bcl-2 seemed to be 'defending' against early relapse.

Gazzaniga et al. suggest that the increase in the level of Bax gene product contributing to the formation of heterodimers with Bcl-2 protein or Bax homodimers, may be able to repair the apoptotic removal of tumoral cells, therefore preventing the malignant growth and metastatic progression of bladder cancer (17).

On the contrary, in those tumors where the Bax gene is less expressed, a higher level of Bcl-2, resulting in a prevalence of Bcl-2 complexes, would be able to block the apoptotic pathways, thus causing cancer progression. This fact contrasts with the hypothesis that the relative ratio between Bcl-2 and Bax proteins has the capability of controlling the susceptibility of cells to death stimuli. Actually, the interactions of several molecular modifications are known for bladder cancer, as similar to other multifactorial diseases (17,18) .

Since normal bladder tissues do not express Bcl-2, one can suppose that altered expression of Bcl-2, and the following block of apoptotic pathways, may be the first step in bladder carcinogenesis. According to this, the intracellular balance between Bcl-2 and Bax may determine the prospect of cancer progression and partly predict the clinical progression of tumoral diseases.

The occurrence of local relapses in superficial bladder cancer is one of the major problems in the clinical management of this tumor. In fact, it is widely accepted that stage and grade are often unable to predict the local progression of Ta-T1 low grade bladder tumors (18) and that 50% of patients with superficial disease have local relapses in the first year of follow-up (19). That is why in 1996 the European Organization for Research and Treatment of Cancer and Medical Research Council also determined the utility of prophylactic treatment in stage Ta-T1-G1 bladder cancer patients, since it gives an advantage in terms of duration of disease-free interval (20). Considering this, the search for a panel of molecular markers could be useful in the identification of patients with a higher risk of relapse. In our series of patients, the combination of a high Bcl-2/Bax ratio expression seems to identify some patients with shorter relapse-free time.

## Conclusion

Our findings, pointing a role of Bcl-2/Bax expression ratio in the progression of TCC, indicate that this ratio may be used as a significant prognostic indicator for prediction of the clinical outcome for patients with lowgrade bladder cancer.

Thus, we suggest that the analysis of the Bcl-2/Bax expression ratio in such tumors may be important as it can contribute to the isolation, follow-up and treatment of "highrisk" patients in the first stages of disease.

Moreover, it is needed to investigate the role that products of other members of the Bcl-2 family and p53 protein may play in the progression of bladder cancer.
